# Acute exacerbation of idiopathic pulmonary fibrosis: lessons learned from acute respiratory distress syndrome?

**DOI:** 10.1186/s13054-018-2002-4

**Published:** 2018-03-23

**Authors:** Alessandro Marchioni, Roberto Tonelli, Lorenzo Ball, Riccardo Fantini, Ivana Castaniere, Stefania Cerri, Fabrizio Luppi, Mario Malerba, Paolo Pelosi, Enrico Clini

**Affiliations:** 1 University Hospital of Modena, Pneumology Unit and Center for Rare Lung Diseases, Department of Medical and Surgical Sciences, University of Modena Reggio Emilia, Modena, Italy; 2 San Martino Policlinico Hospital, IRCCS for Oncology, Department of Surgical Sciences and Integrated Diagnostics, University of Genoa, Genoa, Italy; 3 San Andrea Hospital—ASL Vercelli, Pneumology Unit, Department of Translational Medicine, University of Piemonte Orientale, Novara, Italy

**Keywords:** Idiopathic pulmonary fibrosis, Mechanical ventilation, Acute respiratory distress syndrome, Respiratory failure, Diffuse alveolar damage

## Abstract

Idiopathic pulmonary fibrosis (IPF) is a fibrotic lung disease characterized by progressive loss of lung function and poor prognosis. The so-called acute exacerbation of IPF (AE-IPF) may lead to severe hypoxemia requiring mechanical ventilation in the intensive care unit (ICU). AE-IPF shares several pathophysiological features with acute respiratory distress syndrome (ARDS), a very severe condition commonly treated in this setting.

A review of the literature has been conducted to underline similarities and differences in the management of patients with AE-IPF and ARDS.

During AE-IPF, diffuse alveolar damage and massive loss of aeration occurs, similar to what is observed in patients with ARDS. Differently from ARDS, no studies have yet concluded on the optimal ventilatory strategy and management in AE-IPF patients admitted to the ICU. Notwithstanding, a protective ventilation strategy with low tidal volume and low driving pressure could be recommended similarly to ARDS. The beneficial effect of high levels of positive end-expiratory pressure and prone positioning has still to be elucidated in AE-IPF patients, as well as the precise role of other types of respiratory assistance (e.g., extracorporeal membrane oxygenation) or innovative therapies (e.g., polymyxin-B direct hemoperfusion). The use of systemic drugs such as steroids or immunosuppressive agents in AE-IPF is controversial and potentially associated with an increased risk of serious adverse reactions.

Common pathophysiological abnormalities and similar clinical needs suggest translating to AE-IPF the lessons learned from the management of ARDS patients. Studies focused on specific therapeutic strategies during AE-IPF are warranted.

## Background

Idiopathic pulmonary fibrosis (IPF) is a chronic disease of unknown etiology characterized by a deterioration of the structure of lung parenchyma, thus resulting in a progressive decline of respiratory function and early mortality [1].

In the course of the disease, patients suffering from IPF may develop acute exacerbations of respiratory function impairment, referred to as AE-IPF [2], which can lead to severe acute hypoxemic respiratory failure, sharing common features with acute respiratory distress syndrome (ARDS).

Although patients with AE-IPF receive mechanical ventilation in the intensive care unit (ICU), few studies report their inhospital mortality risk compared to ARDS [3]. Moreover, while an approach with protective mechanical ventilation at low tidal volume is essential to improve survival in ARDS, the least harmful mechanical ventilation strategy is not yet fully elucidated in AE-IPF patients. Table 1 presents a comparison of diagnostic criteria for AE-IPF and ARDS, highlighting a clear overlap between the two conditions.

**Table 1:** Ultimate definition and diagnostic criteria of AE-IPF and ARDS

AE-IPF	ARDS
*Revised definition*	*Berlin definition*
An acute, clinically significant respiratory deterioration characterized by evidence of new widespread alveolar abnormality	A type of acute diffuse, inflammatory lung injury, leading to increased pulmonary vascular permeability, increased lung weight, and loss of aerated lung tissue. The clinical hallmarks are hypoxemia and bilateral radiographic opacities, associated with increased venous admixture, increased physiological dead space, and decreased lung compliance. The morphological hallmark of the acute phase is diffuse alveolar damage (i.e., edema, inflammation, hyaline membrane, or hemorrhage)
*Diagnostic criteria*	*Definition criteria*
Previous or concurrent diagnosis of IPF	
Acute worsening or development of dyspnea typically < 1 month in duration	Onset of lung injury within 1 week of a known clinical insult or new or worsening respiratory symptoms
Computed tomography with new bilateral ground-glass opacity and/or consolidation superimposed on a background pattern consistent with usual interstitial pneumonia pattern	Bilateral opacities—not fully explained by effusions, lobar/lung collapse, or nodules
Deterioration not fully explained by cardiac failure or fluid overload	Respiratory failure not fully explained by cardiac failure or fluid overload

*AE-IPF* acute exacerbation of idiopathic pulmonary fibrosis, *ARDS* acute respiratory distress syndrome

The purpose of this narrative review is to discuss the pathophysiological similarities and differences between AE-IPF and ARDS and to analyze the evidence on treatments currently proposed for AE-IPF, including mechanical ventilation strategies and other therapies.

## ARDS and AE-IPF: similarities and differences

### Diffuse alveolar damage

The typical pathological feature of AE-IPF is the presence of diffuse alveolar damage (DAD) superimposed on the usual interstitial pneumonia (UIP) pattern [4]. The term DAD was proposed by Katzenstein et al. [5] to describe an aspecific acute reaction of the lung to several different pathogenic *noxae*, including sepsis, pneumonia, and exposure to high oxygen concentration. DAD is also the histologic hallmark of ARDS, although this feature can only be found at biopsy in about half of patients meeting the clinical criteria for ARDS diagnosis [6]. In this setting, an exudative phase with endothelial and alveolar epithelial injury and cellular exudate and hyaline membrane deposition develops during the first week from onset. In patients with a condition lasting longer than 3 weeks, proliferation of alveolar cell type 2 and fibroblasts with fibrotic deposition then occurs in 2/3 of cases [7]. Data on histological findings of DAD over AE-IPF development are not available, but it is likely that alveolar damage in survivors may lead to a proliferative reaction with further lung fibrosis.

A retrospective analysis in patients with ARDS who underwent open lung biopsy showed a significant increase in hospital mortality in patients with DAD compared to those without DAD (71.9% vs 45.5%) [8]. Despite this, mortality in patients with ARDS developing DAD is still lower than that reported in AE-IPF patients undergoing invasive mechanical ventilation, which can reach 95% [9]. It is likely that, in patients with IPF, the greater susceptibility of the lung to develop ventilator-induced lung injury (VILI), the impaired ability to repair the acute alveolar damage, and the older age of patients might play a role to explain the worse mortality rate.

Some evidence shows that the clinical features and prognosis of AE-IPF according to the mentioned definition are very similar to the exacerbation of IPF with known cause such as pneumonia or aspiration [10]. Since exacerbation in both idiopathic and non-idiopathic disease results in the development of DAD superimposed on the UIP pattern, a revision of the definition of AE-IPF was proposed focusing on pathobiology. Thus, AE-IPF has been defined as the occurrence of clinical and radiological acute lung injury with DAD regardless of the trigger condition [11, 12].

### Lung inflammation

During the course of AE-IPF, the percentage of neutrophils in bronchoalveolar lavage (BAL) fluid is significantly increased compared with stable chronic IPF, while lymphocytes and macrophages are reduced [13]. This cell pattern is similar to that found in patients with ARDS, which suggests a common inflammatory pathway.

In AE-IPF, the upregulation of M1 macrophage activation chemokines such as IL-8 and CXCL1 results in neutrophil chemoattraction. Interestingly, in animal models, the increased expression of CXC chemokines and their interaction with the CXCR2 receptor are involved in the lung sequestration of neutrophils following mechanical stress due to ventilation, thus suggesting a role in the development of VILI [14]. Furthermore, some studies indicate a relationship between IL-8 overexpression in BAL and the development of ARDS in patients at risk [15]. Acute hypoxia could act as a proinflammatory stimulus leading to a rapid increase of intrapulmonary IL-8, released by alveolar macrophages with attraction of neutrophils and subsequent alveolar and endothelial injury [16].

The alternative M2 macrophage activation pathway was also observed in AE-IPF, playing a determinant role in damage healing [13, 17]. A direct link between injury to type II alveolar epithelial cells and the accumulation of interstitial collagen by M2 pathway activation was reported [18], which could stimulate repair by fibroblast proliferation and epithelial–mesenchymal transition. This repair process, however, appears to fail in AE-IPF, thus resulting in persistent M2 pathway activation and irreversible lung fibrosis [19]. A recent study on lungs of transplanted IPF patients showed that inflammatory infiltration and DAD are even present in IPF with an accelerated functional decline, suggesting that inflammation may play a role in disease progression [20]. Further evidence that the cytokine profile in the rapidly deteriorating IPF patient appears predominantly proinflammatory rather than profibrotic, approximating that of ARDS of any etiology rather than an accelerated intrinsic fibrotic process, has been provided by Papiris et al. [21].

**Fig. 1:**
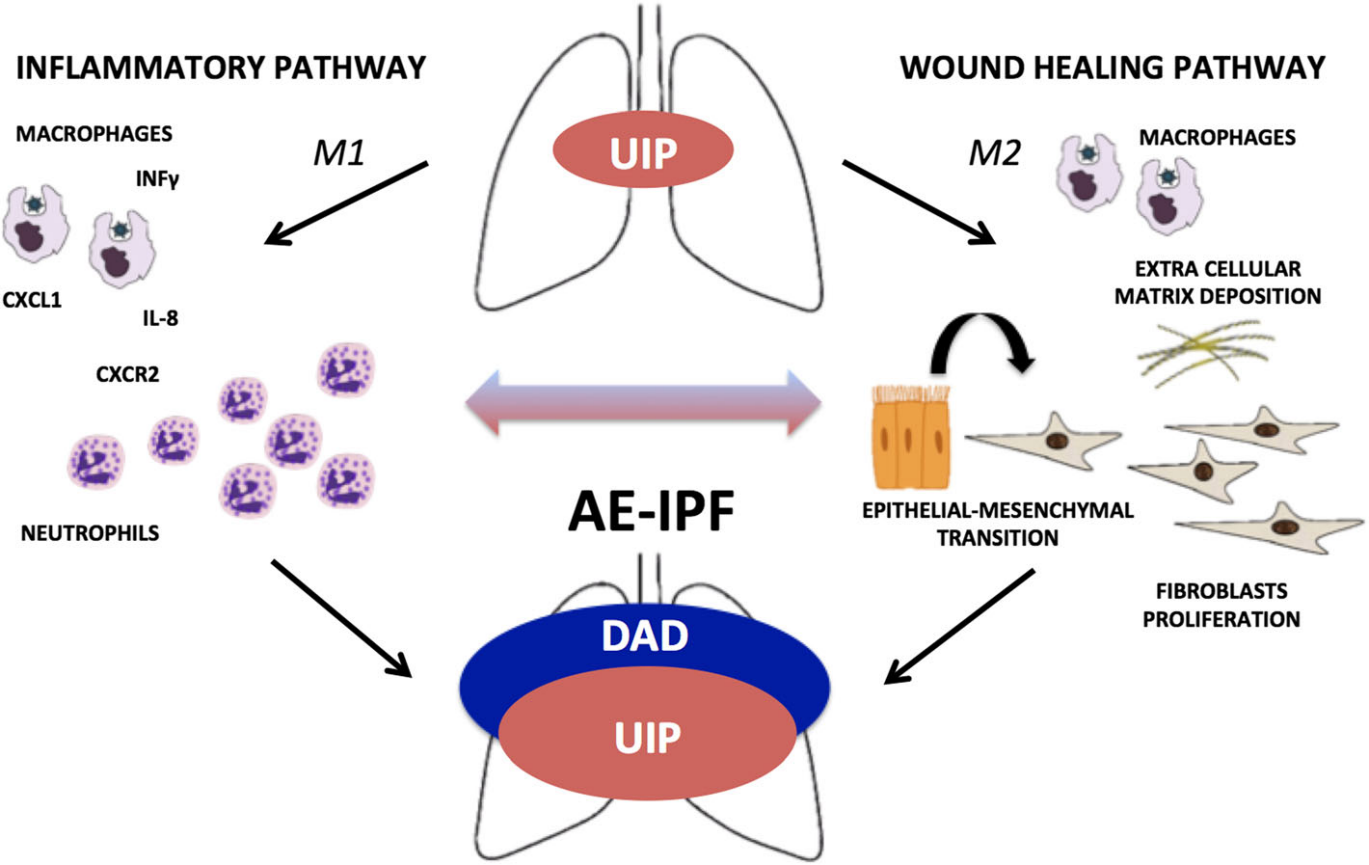
During AE-IPF, lung inflammation is driven by upregulation of macrophage activation pathways. M1 pathway classically activated by Th1 cytokines (IFN-γ) leads to increased IL-8 and CXCL1 expression and neutrophil recruitment though CXCR2 receptor. M2 pathway activated by type II alveolar epithelial cell injury might perpetuate lung fibrosis boosting collagen deposition, fibroblast proliferation, and epithelial–mesenchymal transition. AE-IPF acute exacerbation of idiopathic pulmonary fibrosis, DAD diffuse alveolar damage, IL interleukin, INF interferon

Therefore, both ARDS and AE-IPF share an overexpression of proinflammatory cytokines produced by alveolar macrophages with chemotaxis of neutrophils. However, overexpression of anti-inflammatory M2 cytokines with a profibrotic role is simultaneously present only in AE-IPF (see Fig. 1).

### Respiratory mechanics and ventilator-induced lung injury

In patients with ARDS, several studies have documented changes in lung mechanics, describing the role of mechanical ventilation in the development of VILI and the consequent increased risk of death [22]. Much less is known concerning the impact of VILI on mortality in AE-IPF patients. Despite this, the aforementioned shared pathophysiological features provide a rationale for translating to AE-IPF the lessons derived from ventilatory management in ARDS patients. Moreover, VILI can occur also in patients without ARDS [23], even in those with healthy lungs, providing a stringent rationale for developing lung-protective strategies for all indications of mechanical ventilation, from the operating room to any critically ill patient.

Over the past 20 years, following the awareness that VILI can highly contribute to mortality, the goal of mechanical ventilation in ARDS has changed from improving gas exchange to protecting the lung from the damage induced by mechanical ventilation [24, 25]. Unphysiological stress (distension of force per unit area as defined by the transpulmonary pressure reached at end inspiration) and strain (deformation, namely the ratio of tidal volume to the end-expiratory lung volume, V_T_/EELV) applied to the lung tissue are the physical forces responsible for the development of VILI [26, 27].

Defining a threshold of safety for stress and strain remains a challenge [28]. Transpulmonary pressure measurement (the difference between airway and pleural pressure, estimated assuming that the pleural pressure approximates the esophageal pressure) [29] could add information for patients with AE-IPF, who also have increased chest wall stiffness, as is the case of morbidly obese patients, in addition to the expected increase in lung elastance.

Since stress and strain are not measured routinely, the plateau pressure and the tidal volume are considered surrogates of stress and strain, respectively, and are monitored closely in clinical practice when setting the ventilator in patients with ARDS. Currently, it is recommended to maintain an airway plateau pressure below 30 cmH_2_O and set a V_T_ less than 6 ml/kg of predicted body weight [24]. Notwithstanding, the plateau pressure and V_T_ are parameters that are easy to monitor during mechanical ventilation but inadequate to truly represent the stress and the strain applied to the lung [28].

Recently, interest has grown toward the variation of airway pressure achieved during tidal breath (namely the airway driving pressure, ΔP). ΔP equals plateau pressure minus positive end-expiratory pressure and can be considered the *dynamic stress*, representing the ratio between V_T_ and the compliance of the respiratory system. It is reasonable to assume that compliance and end-expiratory lung volume (EELV), both being associated with the severity of lung injury, are correlated: under this assumption, ΔP would also reflect V_T_/EELV (i.e., strain). Thus, the ΔP of the respiratory system or the lung represents a simple and promising tool at the bedside to monitor the injury caused by ventilation.

The transpulmonary pressure (∆P_L_) and the absolute level of transpulmonary pressure at end inspiration depend on the ratio between lung elastance (E_L_) and the total elastance of the respiratory system (E_TOT_ = E_L_ + E_CW_) according to the following equation [30]:

$$ {\mathbf{P}}_{\mathbf{L}}={\mathbf{P}\mathbf{aw}}^{\ast }\ {\mathbf{E}}_{\mathbf{L}}/{\mathbf{E}}_{\mathbf{TOT}} $$

This ratio is normally 0.5 at functional residual capacity. In patients with ARDS, acute lung injury is known to cause a significant increase in total elastance secondary to the increase in lung elastance, but possibly also to the chest wall elastance [31]. The E_L_/E_TOT_ ratio may vary substantially and ranges from 0.2 to 0.8 [32]. This means that patients with the same plateau pressure can have harmful or safe transpulmonary pressures [33].

Another aspect that must be considered in ARDS is that the inhomogeneity of the lung might act regionally as a stress raiser, increasing the pressure applied to patent respiratory units surrounded by nonaerated units [34].

Finally, in the very last years, the concepts of mechanical energy [35] and power [36] have been introduced to describe VILI in terms of energy transfer from the ventilator to the respiratory system. These concepts still require extensive validation, but have the advantage of trying to combine all of the different aspects of VILI into a single parameter.

All of these features and concepts specifically refer to ARDS, and much less is known for AE-IPF. In a single study evaluating the respiratory mechanics of mechanically ventilated patients with end-stage IPF [37], a marked increase in the elastance of the respiratory system (51 cmH_2_O/L) was reported, mainly due to an abnormal lung elastance (46 cmH_2_O/L) with a normal chest wall elastance (5 cmH_2_O/L) and an E_L_/E_TOT_ ratio around 0.9: In this case, the application of a plateau pressure of 30 cmH_2_O at a PEEP of 4 cmH_2_O, which are elevated pressures often seen in AE-IPF patients, causes a ∆P of 30 – 4 = 26 cmH_2_O, with an absolute end-inspiratory transpulmonary pressure of 30 × 0.9 = 27 cmH_2_O. Both values are above acceptable levels. If feasible in terms of gas exchange, a reduction of plateau pressure and driving pressure should be warranted.

Furthermore, alveolar collapse and consolidation, that are responsible for permanent derecruitment, are present in IPF and do not improve with the application of positive pressure to the airways. Collapse induration is characterized by septal wall thickening and alveolar epithelial hyperplasia, with obliteration of alveoli due to enlargement and overgrowth of epithelial type II cells [38]. It is therefore easy to understand that the application of a high PEEP to these lungs cannot result in recruitment of hypoventilated areas, but can facilitate overinflation in the spared areas of the lung, with further deterioration of its mechanical properties. In agreement with this concept, one study showed that a high PEEP level in patients with interstitial lung disease undergoing mechanical ventilation is independently associated with increased mortality [39].

**Fig. 2:**
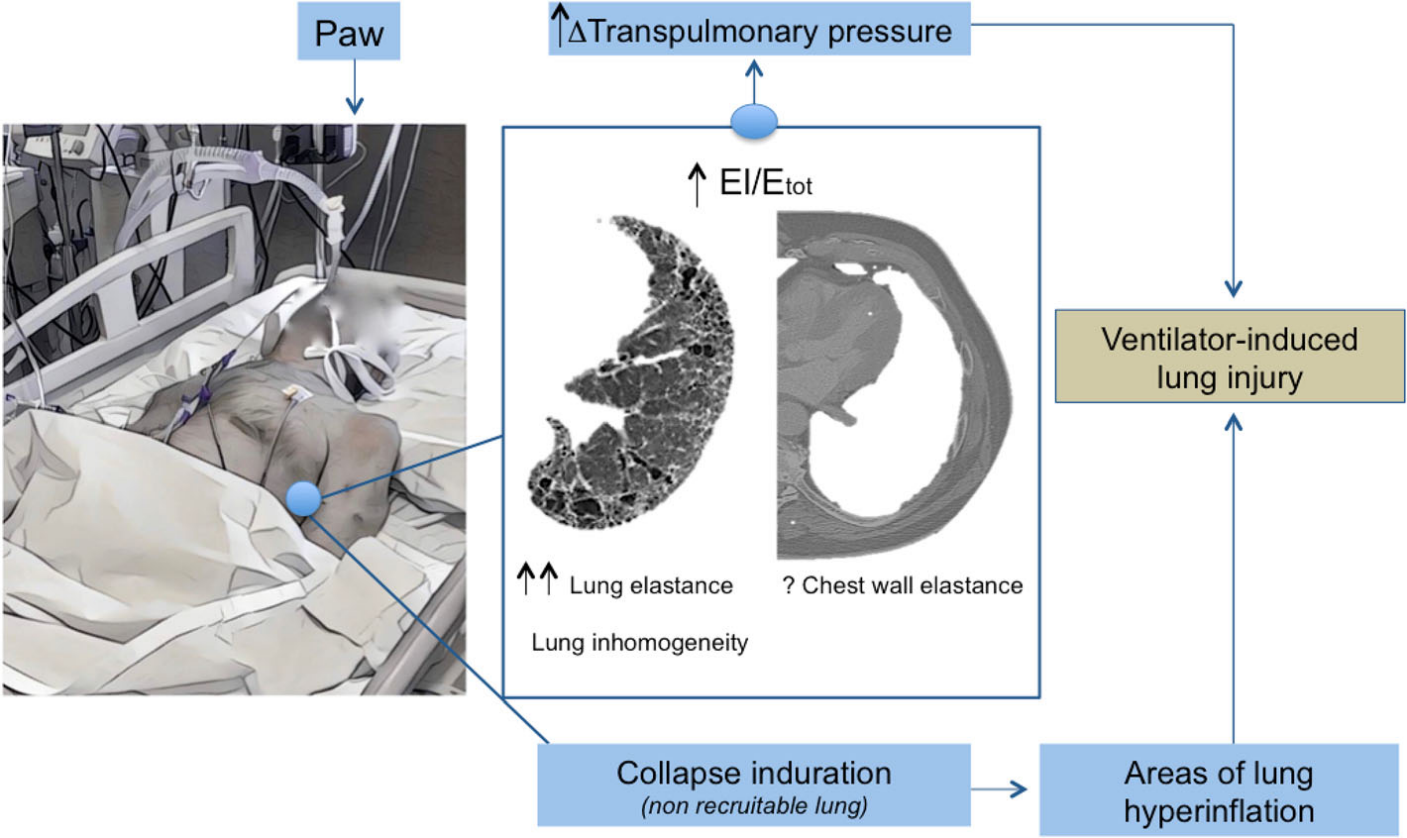
Mechanisms of ventilation-induced lung injury in patients with AE-IPF. El elastance of lung, Etot elastance of respiratory system, Paw airway pressure

Therefore, despite some similarities with ARDS, the lung in AE-IPF is characterized by some unique pathophysiological properties (i.e., collapse induration areas, elevated lung elastance, high inhomogeneity) that might make it more susceptible to VILI. Figure 2 summarizes the mechanisms leading to VILI in AE-IPF.

## Respiratory assistance

Few studies have evaluated the outcome of patients with AE-IPF receiving mechanical ventilation in the ICU, and in addition they all share important limitations (see Table 2): single-centered and retrospective analysis; limited number of patients included; unclear or unreported mode of ventilation and setting; and heterogeneous use of drugs [4, 39–42]. Overall, the available data are consistent in stating that invasive mechanical ventilation cannot significantly modify the poor prognosis of these patients [9]. Despite the aforementioned studies being performed before the extensive use of protective mechanical ventilation to prevent VILI, the American Thoracic Society guidelines on IPF recently recommended the use of mechanical ventilation only in a few selected patients developing severe AE [1]. More recently, a multicenter retrospective study in the United States documented an overall mortality rate of 51% in a large group of mechanically ventilated AE-IPF patients [3], still higher than that reported in severe ARDS (about 40%) [43] but lower than that described previously. Thus, one could speculate that advances leading to a better ventilator management and outcome of patients with ARDS may have also positively influenced the outcomes in ventilated AE-IPF.

**Table 2:** Studies investigating the use of mechanical ventilation in patients experiencing AE-IPF and major outcomes

Study	Time frame	*N*	MV	AE-IPF	NIV	Ventilator setting	ICU mortality	Hospital mortality
Molina-Molina et al. [106]	1986–2002	14	14	NR	NR	NR	NR	85% (11/13)
Nava and Rubini [37]	1998	7	7	NR	0	V_T_ 8.3 ml/kg	86% (6/7)	NR
Stern et al. [107]	1991–1999	23	23	16	NR	V_T_ 8–13 ml/kg	96% (22/23)	96% (22/23)
Blivet et al. [108]	1989–1998	15	15	6	5	NR	73% (11/15)	87% (13/15)
Saydain et al. [109]	1995–2000	38	19	15	7	NR	68% (13/19)	61% (23/38)
Fumeaux et al. [110]	1996–2001	14^a^	14	NR	11	V_T_ 7–9 ml/kg	100% (14/14)	100% (14/14)
Al-Hameed and Sharma [80]	1998–2000	25	25	25	3	PEEP 7 cmH_2_O	84% (21/25)	96% (24/25)
Kim et al. [4]	1990–2003	10	9	9	NR	NR	78% (7/9)	78% (7/9)
Pitsiou et al. [30]	2001–2005	12	12	NR	NR	NR	100 (12/12)	100% (12/12)
Rangappa and Moran [112]	1996–2006	24	19	8	NR	NR	67% (16/24)	92% (22/24)
Fernandez-Pèrez et al. [113]	2002–2006	30	30	NR	NR	V_T_ 7–8 ml/kg	NR	60% (18/30)
Mollica et al. [114]	2000–2007	34	34	22	19	V_T_ 7.5 ml/kg or PS/PEEP 18/7 cmH_2_O	100% IMV, 73% NIV	85% (29/34)
Yokoama et al. [85]	1998–2004	11	11	11	11	CPAP 10 cmH_2_O, PS/PEEP 5/10 cmH_2_O	NR	56% (6/11) (3 months)
Gungor et al. [115]	2000–2007	96	96	NR	28	V_T_ 6-8 ml/kg, PEEP 5–7 cmH_2_O	64% (61/96)	NR
Vianello et al. [116]	2005–2013	18	18	6	18	PEEP 5–8 cmH_2_O	56% (10/18)	NR
Gaudry et al. [117]	2002–2009	22	22	NR	0	V_T_ 5.9 ml/kg, PEEP 7.1 cmH_2_O	67% (17/22)	NR
Aliberti et al. [41]	2004–2009	60	60	24	60	CPAP 8 cmH_2_O, PS/PEEP 5/15	NR	35% (21/60)
Total		453	428	142	162			

*AE-IPF* acute exacerbation of idiopathic pulmonary fibrosis, *MV* mechanical ventilation, *NIV* noninvasive mechanical ventilation, *ICU* intensive care unit, *NR* not reported, *V*_*T*_ tidal volume, *PEEP* positive end-expiratory pressure, *PS* pressure support, *CPAP* continuous positive airway pressure

^a^Three non-IPF

Overall, the inconsistency of data and the lack of extensive evidence still suggest considering ICU admission and respiratory assistance only in selected cases of AE-IPF, mainly based on the following criteria: shorter time from diagnosis, accounting for the fact that average survival is 3 years; younger age and fewer comorbidities; and eligibility and high chances of lung transplantation [44].

Although not yet determined by specific studies, available options to deliver respiratory assistance are as follows: controlled ventilation mode, prone position, assisted ventilation mode and extracorporeal membrane oxygenation (ECMO).

### Controlled ventilation modes

Pressure-controlled or volume-controlled invasive ventilation is the most widely applied mode of respiratory support for AE-IPF. As learned from ARDS, even in AE-IPF the main objective of ventilation should be lung protection, avoiding VILI (see earlier) while ensuring an acceptable, but not necessarily optimal, gas exchange. As reported in the literature, it is reasonable to apply a tidal volume even lower than 6 ml/kg of ideal body weight to target a plateau pressure lower than 30 cmH_2_O [45]. Moreover, these patients require a higher respiratory rate and minute ventilation, due to an increased physiologic dead space, allowing permissive hypercapnia.

Despite the lack of studies about the usage of neuromuscular blockade in IPF patients, we could hypothesize that complete muscle paralysis at the early onset of severe AE could help in reducing the lung stress and strain, and avoiding a deleterious patient–ventilator asynchrony [46]. Positive end-expiratory pressure (PEEP) should be set at low–moderate levels (e.g., 4–6 cmH_2_O), taking into account the intrinsic low recruitability potential, with high risk of hyperinflation. Indeed, poor survival with high end-expiratory pressure applied has been documented in a cohort of patients with interstitial lung disease [47]. An *open lung approach*, with recruitment maneuvers, recently questioned even in the early phase of ARDS [48], has no physiological rationale in AE-IPF, and should therefore be avoided.

In patients with AE-IPF with high plateau pressure, the measurement of esophageal pressure (Pes) as a surrogate marker of pleural pressure may allow one to identify the lung stress and the risk of injury from ventilation by calculating the inspiratory transpulmonary pressure. To date, only experimental models suggest setting the protective mechanical ventilation to a ‘probably safe’ P_L_ level [27], namely below 20 cmH_2_O in homogeneous lungs or below 12 cmH_2_O in inhomogeneous lungs, as is the case in AE-IPF [29].

### Prone position

Prone position (PP) has been used since the 1970s as a rescue therapy for severe hypoxemia in patients with ARDS [48]. An improvement of oxygenation with PP occurs regardless of the cause of ARDS, and it is most evident during the exudative early phase of the disease [49] or when applied early for at least 16 h per day in moderate-to-severe ARDS (PaO_2_/FiO_2_ < 150 mmHg) [50].

Only one study has evaluated the effect of PP on gas exchange in pulmonary fibrosis by comparison with both hydrostatic pulmonary edema and ARDS [51]. In patients with fibrosis, changing the position from supine to prone did not improve oxygenation, while there was an increase of the plateau pressure and a reduction in Crs [52]. Therefore, prone positioning in AE-IPF cannot be recommended.

### Assisted ventilation modes

Spontaneous assisted breathing can have beneficial effects on shunt reduction and improvement in oxygenation, maintaining diaphragmatic tone and increasing dependent lung ventilation, in moderate but not severe acute respiratory failure [53, 54]. Nonetheless, experimental data suggest that spontaneous breathing activity can improve lung function and decrease inflammation in moderately injured lungs [55].

During assisted spontaneous breathing, inspiratory muscle activity leads to negativity of the pleural pressure, and thoracic structures are subject to inward forces. Patients with AE-IPF have a significant hyperactivation of the respiratory drive with a pleural swing that can even reach −30 to −40 cmH_2_O. This means that, during assisted ventilation, this is the major contribution to the total transpulmonary pressure, also when airway pressure is apparently low. Even at comparable flow and volume conditions, spontaneous breathing can be more injurious when patients present a high respiratory drive [56].

The reason for this effect on pulmonary stress depends on several factors. First, airway pressure can fall under end-expiratory pressure during spontaneous breathing, when performing vigorous inspiratory efforts [57]. In this case, pulmonary vessels are subject to negative pressure, with increased transmural vascular pressure, risk of alveolar edema, and progression to VILI. Second, the change in transpulmonary pressure during the respiratory effort occurs inhomogeneously, resulting in a heterogeneous lung expansion without a gain in V_T_ [58, 59]. This phenomenon is related with the *pendelluft* effect*,* namely the fast exchange of gas volume that occurs during strong effort between different regions of the lung before starting V_T_, with deflation of nondependent regions and gas swing toward the dependent regions, which leads to increased local stretch: this regional inflation–deflation pattern is considered one of the causes of injury. Third, patient–ventilator asynchrony may increase the risk of lung injury [58]. Early use of neuromuscular blocking agents in severe hypoxemia (Pa/FIO_2_ < 120 mmHg) may counteract these potentially detrimental effects of assisted breathing, resulting in improved survival.

**Fig. 3:**
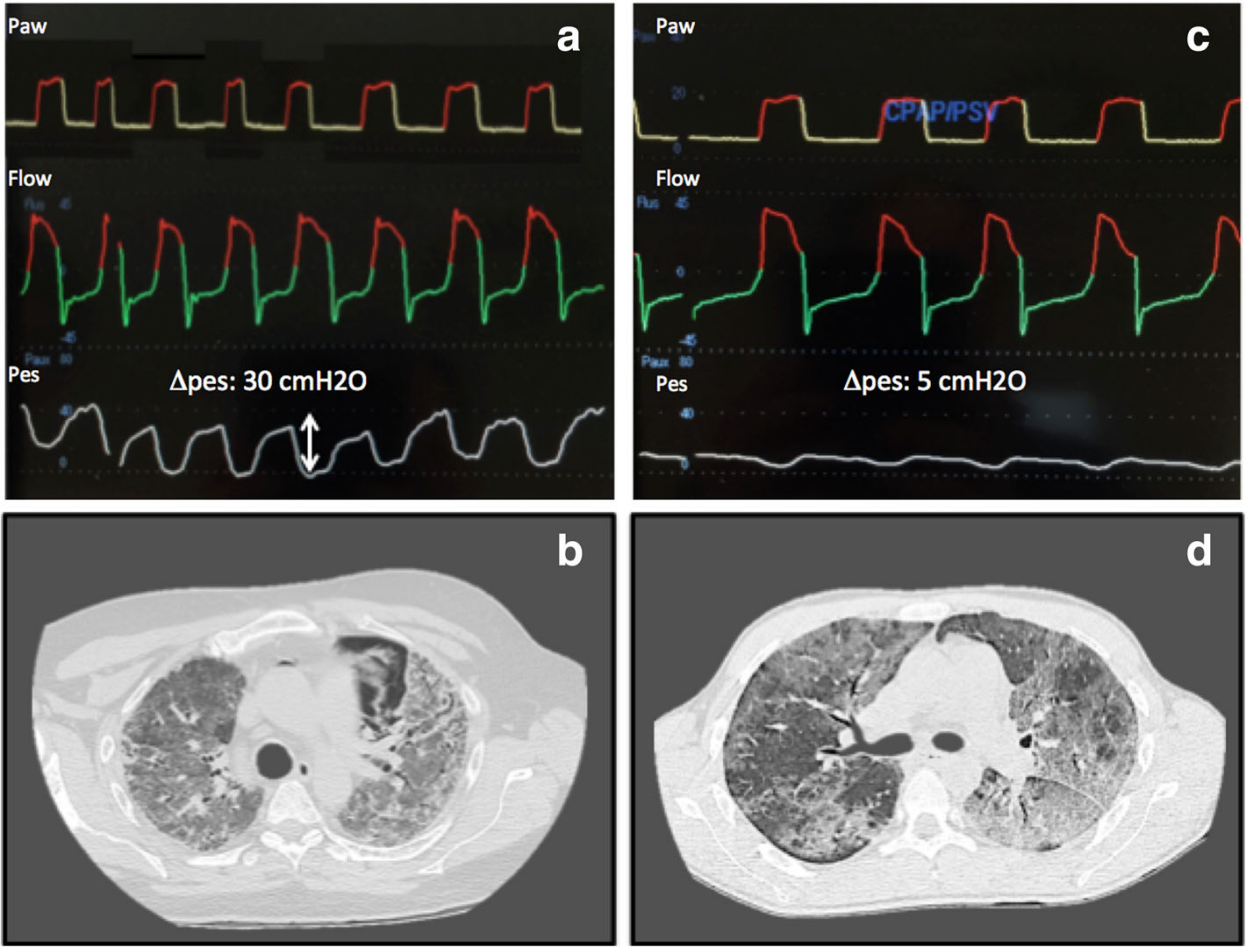
**a** Patient with AE-IPF during assisted spontaneous breathing with end-expiratory positive pressure of 4 cmH_2_O and pressure support of 10 cmH_2_O. Note ΔPes of 30 cmH_2_O due to respiratory drive hyperactivity. **b** Thorax CT scan performed on same patient as (a), showing anterior left pneumothorax probably due to high transpulmonary pressure. Note homogeneous increase of parenchymal density. **c** Patient with AE-IPF during assisted spontaneous breathing with end-expiratory positive pressure of 4 cmH_2_O and pressure support of 10 cmH_2_O. Note ΔPes of 5 cmH_2_O due to normal activation of respiratory drive. **d** Thorax CT scan performed on same patient as (b) showing nonhomogeneous opacities in lung parenchyma. Pes esophageal pressure. Paw airway pressure

In patients with AE-IPF, monitoring the respiratory drive with occlusion pressure (P_01_), esophageal pressure, and V_T_ during spontaneous breathing could therefore be helpful in identifying patients at risk of self-inflicted lung injury (SILI) and to verify favorable changes when invasive pressure support ventilation is applied [56]. Since the respiratory drive is not only affected by the level of pressure support but also by the degree of sedation, use of sedatives could be considered part of a protective ventilation strategy in patients with high respiratory drive. Figure 3 shows mechanical tracing and chest tomography in two patients with AE-IPF subjected to a similar level of pressure support ventilation but with different activation of respiratory drive, as reflected by the different esophageal pressure swing and pulmonary stress.

Noninvasive ventilation (NIV) is a method of spontaneous breathing support not requiring endotracheal intubation, potentially reducing the risk of ventilator-associated pneumonia (VAP). Retrospective studies that have analyzed the effectiveness of NIV in AE-IPF reported a mortality rate between 45 and 75%, always related to the worsening of respiratory failure [38–40]. Overall, the decision to start NIV was based on the occurrence of moderate-to-severe dyspnea, respiratory rate above 30 breaths/min, signs of increased work of breathing, and/or PaO_2_/FIO_2_ ratio below 250 mmHg. In most of these studies, NIV was initially delivered continuously in the first 24–48 h and then weaned progressively to longer unassisted intervals, according to the clinical conditions and gas exchange.

More recently, an observational study on a large cohort of patients with AE-IPF who underwent mechanical ventilation showed a lower mortality rate when NIV was applied (30.9%) as compared to conventional mechanical ventilation (51.6%) [3]. At least theoretically, the survival advantage could be due to the early application of NIV in patients with less severe general conditions, and the ability of preventing VAP.

Also, high-flow oxygen delivered through nasal cannulae (HFNO) has proven efficacy in the management of nonhypercapnic acute respiratory failure [50]. To date, we are not aware of any randomized trial evaluating the effects of HFNO in patients with AE-IPF. Only a case series by Horio et al. [60] showed that, when used in IPF patients during AE, HFNO is well tolerated and associated with increased ventilation efficiency, decreased respiratory rate, and reduced work of breathing. However, the potential effectiveness of HFNO should be carefully assessed in this specific subset of hypoxic patients with particular reference to the potential enhancement of fibrotic damage in the lungs following long-term exposure to high concentrations of oxygen.

### Extracorporeal membrane oxygenation

Extracorporeal life support is a salvage strategy increasingly applied in ARDS with severe hypoxemia. Venovenous extracorporeal membrane oxygenation (ECMO) is able to provide adequate gas exchange beyond mechanical ventilation, and can potentially reduce the injurious effects of positive pressure ventilation [61]. The best strategy to ventilate patients receiving ECMO is still debated; however, also in this setting higher driving pressure was associated with increased inhospital mortality [62].

There is evidence that patients who received mechanical ventilation in the pretransplant period have a significantly higher posttransplant mortality than nonventilated patients, suggesting that a bridge treatment with ECMO should be provided as early as possible [63]. Indeed, ECMO was used in awake nonintubated patients to preserve the tone of respiratory muscles, as well as to achieve early mobilization and to facilitate posttransplant weaning [64]. A review including 14 studies evaluated patients with mixed diseases bridged to transplant with ECMO as an alternative to invasive mechanical ventilation [65], and showed better 6-month survival compared with mechanical ventilation (62% and 35%, respectively). In this analysis, the IPF population ranged from 27 to 62% of patients, and diagnosis of pulmonary fibrosis was not associated with worse survival [65].

Thus, it can be argued that ECMO might be a promising strategy to bridge lung transplantation in severe patients developing AE-IPF. Notwithstanding, reduced availability and high costs may limit its use in this condition.

## Other therapies

### Steroids and immunosuppressive agents

At present, no randomized controlled trials on drug treatments in AE-IPF are available; therefore, recommendations of international consensus are based on weak evidence, as is the case for systemic glucocorticoids (GC) [1].

Considering again ARDS as a model, perpetuated DAD can be assumed as a dysregulated systemic and pulmonary inflammatory condition, where massive elevation of inflammatory cytokines in blood and BAL fluid correlates with a worse prognosis [66].

GCs are able to block nuclear translocation of NF-κB, the main pathway of inflammatory cytokine synthesis, through their interaction with the glucocorticoid receptor (GR). Despite this rationale, the use of steroids in ARDS is not recommended routinely, as clinical trials have demonstrated improvements in oxygenation and lung mechanics but not in survival [67].

Studies on the correlation between systemic inflammation and prognosis in AE-IPF are lacking. However, one study in IPF showed that ST2 serum levels, a protein expressed in T-helper type 2 cells and induced by proinflammatory stimuli, were higher during AE compared to the stable phase or in healthy controls, suggesting that systemic inflammation is a hallmark of AE-IPF [68], thus opening a potential role for anti-inflammatory drug-based therapy. Notwithstanding, retrospective data derived from AE-IPF patients treated with steroids alone did not show any reduction in mortality rate over the short term (55% in 65 patients treated with methylprednisolone ≥ 500 mg/day or prednisolone both ≥ 0.5 or ≤ 0.5 mg/kg) and the long term (82% at 3 months in 11 patients who received methylprednisolone 1 g/day for 3 days) [69, 70].

Guidelines in France also support the use of intravenous cyclophosphamide, in addition to steroids, as an immunosuppressive agent [71]. A retrospective study in 10 patients with AE-IPF treated with methylprednisolone (1000 mg on days 1–3) followed by cyclophosphamide infusion (500 mg on day 4, then increased by 200 mg every 2 weeks up to 1500 mg) showed 50% survival at 3 months. On the other hand, other small retrospective studies did not show any outcome improvement when using such combination therapy in the same subjects [70, 71].

Moreover, despite the use of many other cytotoxic agents (e.g., azathioprine, cyclosporine A, tacrolimus) being reported anecdotally in other case series of AE-IPF, there is no robust evidence to suggest their use [72]. Finally, some authors have proposed the nonsteroid approach, consisting of immunosuppression cessation (if any), best supportive care, broad-spectrum antimicrobials, and thorough evaluation to detect reversible causes of deterioration [73].

Table 3 summarizes the drugs currently under investigation as a preventative measure for AE-IPF, notwithstanding that the quality of evidence is still limited.

**Table 3:** Pharmacological therapies for AE-IPF, currently proposed or under investigation

Therapy	Study
Nintedanib (preventive therapy)	Richeldi et al., 2011 [74]; Richeldi et al., 2014 [75]
Pirfenidone (preventative therapy)	Azuma et al., 2005 [76]; Taniguchi et al., 2010 [77]
Anti-acid therapy (preventative therapy)	Lee et al., 2013 [78]
Corticosteroid monotherapy	Akira et al., 1997 [79]; Al-Hameed and Sharma, 2004 [80]; Suzuki et al., 2011 [81]; Tachikawa et al., 2012 [82]
Cyclophosphamide	Akira et al., 2008 [83]; Fujimoto et al., 2012 [84]; Tachikawa et al., 2012 [82]; Yokoyama et al., 2010 [85]
Cyclosporine	Homma et al., 2005 [86]; Inase et al., 2003 [87]; Sakamoto, et al., 2010 [88]; Fujimoto et al., 2012 [84]; Yokoyama et al., 2010 [85]
Polymyxin-B immobilized fiber column hemoperfusion	Abe et al., 2011 [89]; Abe et al., 2012 [90]; Oishi et al., 2013 [91]; Seo et al., 2006 [92]; Tachibana et al., 2011 [93]
Rituximab, plasma exchange, and intravenous immunoglobulin	Donahoe et al., 2015 [94]
Tacrolimus	Horita et al., 2011 [95]
Thrombomodulin	Kataoka et al., 2015 [96]; Tsushima et al., 2014 [97]; Isshiki et al., 2015 [98]
Cessation of immunosuppression, best supportive care, broad-spectrum antimicrobials: “nonsteroid approach”	Papiris et al., 2015 [73]

*AE-IPF* acute exacerbation of idiopathic pulmonary fibrosis

### Polymyxin-B direct hemoperfusion

Polymyxin-B (PMX-B) is a polypeptide antibiotic with bactericidal activity toward Gram-negative bacteria that binds circulating endotoxin [99]. In patients with severe sepsis, septic shock, or refractory shock, the use of a PMX-B direct hemoperfusion (PMX-B DHP) cartridge has proven high efficacy in reducing the level of circulating endotoxin, removing blood cytokines and activated neutrophils, and preventing the endothelial damage caused by reactive oxygen species (ROS) [100]. In patients with ARDS developing DAD, PMX-B DHP showed a significant improvement in blood oxygenation [101]. The use of PMX-B DHP in AE-IPF was first investigated in Japan with an open-label pilot study, followed by two case reports that assessed the safety of the procedure [92]. Retrospective studies reported survival rates of 47%, 32% and 26% at 1, 2 and 3 months, respectively, after PMX-B DHP in patients with AE-IPF [93] and significant improvement in the PaO_2_/FIO_2_ ratio in patients with acute exacerbation of interstitial pneumonia of different etiology [90].

Enomoto et al. [102] compared the survival rates of 31 patients with AE-IPF, 14 of which were treated with PMX-B DHP. They described a 1-year survival rate significantly higher in patients receiving PMX-DHP B compared with those under supportive care plus steroids alone (48.2% vs 5.9%, respectively). Compared with controls of similar severity, patients with severe underlying disease, identified by a GAP index score of 2 or 3, showed a 65% risk reduction in mortality following PMX-B DHP [102].

### Lung transplantation

In end-stage IPF, lung transplantation may offer better life expectancy with an overall 5-year survival rate around 50% [103]. Patients with AE-IPF already included in the waiting list should then be admitted to intensive care and bridged to ECMO as soon as possible. In some countries, an emergency list for transplantation is reserved for patients aged younger than 50 years who are admitted to the ICU due to a rapid deterioration of their disease, or requiring any respiratory assistance. The outcome of urgent pulmonary transplantation showed an acceptable survival rate (67% and 59% at 1 and 3 years, respectively) but was greater when compared with elective surgery [104]. High SAPS score (> 24), the need for ECMO, and huge elevation of serum procalcitonin were associated with a poor outcome in these candidates [105].

## Conclusions

AE-IPF shares several pathophysiological features with ARDS, and while the optimal ventilation strategy in these patients has not yet been defined, the extreme fragility of fibrotic lungs suggests adopting a protective ventilation strategy, which seems to positively impact inhospital survival. NIV should only be considered as an early measure, while monitoring the level of respiratory drive activation. ECMO has a role to bridge lung transplantation in severe patients with AE-IPF, but should be started early. Systemic steroids and immunosuppressive agents provide no clear evidence on their ability to change prognosis in AE-IPF.

Taking all of the available evidence into account, it seems that applying the lesson so far learned from ARDS would be the best option to optimally manage AE-IPF as a critical clinical condition affecting the lungs.

## Abbreviations

AE-IPF: Acute exacerbation of IPF
ARDS: Acute respiratory distress syndrome
Crs: Compliance of the respiratory system
DAD: Diffuse alveolar damage
ECMO: Extracorporeal membrane oxygenation
E_L_: Elastance of the lung
E_TOT_: Elastance of the respiratory system
GC: Glucocorticoid
GR: Glucocorticoid receptor
IPF: Idiopathic pulmonary fibrosis
NIV: Non-invasive mechanical ventilation
Pes: Esophageal pressure
P_L_: Transpulmonary pressure
PMX-B: Polymyxin-B
PMX-DHP: Polymyxin-B direct hemoperfusion
SILI: Self-inflicted lung injury
UIP: Usual interstitial pneumonia
VAP: Ventilator-associated pneumonia
VILI: Ventilator-induced lung injury
V_T_: Tidal volume
ΔP: Driving pressure

## References

[1] Raghu G, Collard HR, Egan JJ, Martinez FJ, Behr J, Brown KK, Colby TV, Cordier JF, Flaherty KR, Lasky JA, Lynch DA, Ryu JH, Swigris JJ, Wells AU, Ancochea J, Bouros D, Carvalho C, Costabel U, Ebina M, Hansell DM, Johkoh T, Kim DS, King TE, Kondoh Y, Myers J, Müller NL, Nicholson AG, Richeldi L, Selman M, Dudden RF, Griss BS, Protzko SL, Schünemann HJ, ATS/ERS/JRS/ALAT Committee on Idiopathic Pulmonary Fibrosis (2011). An official ATS/ERS/JRS/ALAT statement: idiopathic pulmonary fibrosis: evidence-based guidelines for diagnosis and management. Am J Respir Crit Care Med.

[2] Collard HR, Moore BB, Flaherty KR, Brown KK, Kaner RJ, King TE, Lasky JA, Loyd JE, Noth I, Olman MA, Raghu G, Roman J, Ryu JH, Zisman DA, Hunninghake GW, Colby TV, Egan JJ, Hansell DM, Johkoh T, Kaminski N, Kim DS, Kondoh Y, Lynch DA, Müller-Quernheim J, Myers JL, Nicholson AG, Selman M, Toews GB, Wells AU, Martinez FJ (2007). Idiopathic Pulmonary Fibrosis Clinical Research Network Investigators. Acute exacerbations of idiopathic pulmonary fibrosis. Am J Respir Crit Care Med.

[3] Rush B, Wiskar K, Berger L, Griesdale D (2016). The use of mechanical ventilation in patients with idiopathic pulmonary fibrosis in the United States: a nationwide retrospective cohort analysis. Respir Med.

[4] Kim DS, Park JH, Park BK, Lee JS, Nicholson AG, Colby T (2006). Acute exacerbation of idiopathic pulmonary fibrosis: frequency and clinical features. Eur Respir J.

[5] Katzenstein AL, Bloor CM, Leibow AA (1976). Diffuse alveolar damage—the role of oxygen, shock, and related factors. A review Am J Pathol.

[6] Cardinal-Fernández P, Bajwa EK, Dominguez-Calvo A, Menéndez JM, Papazian L, Thompson BT (2016). The presence of diffuse alveolar damage on open lung biopsy is associated with mortality in patients with acute respiratory distress syndrome: a systematic review and meta-analysis. Chest.

[7] Thille AW, Esteban A, Fernández-Segoviano P, Rodriguez JM, Aramburu JA, Vargas-Errázuriz P, Martín-Pellicer A, Lorente JA, Frutos-Vivar F (2013). Chronology of histological lesions in acute respiratory distress syndrome with diffuse alveolar damage: a prospective cohort study of clinical autopsies. Lancet Respir Med.

[8] Kao KC, Hu HC, Chang CH, Hung CY, Chiu LC, Li SH, Lin SW, Chuang LP, Wang CW, Li LF, Chen NH, Yang CT, Huang CC, Tsai YH (2015). Diffuse alveolar damage associated mortality in selected acute respiratory distress syndrome patients with open lung biopsy. Crit Care.

[9] Mallick S (2008). Outcome of patients with idiopathic pulmonary fibrosis (IPF) ventilated in intensive care unit. Respir Med.

[10] Collard HR, Yow E, Richeldi L, Anstrom KJ, Glazer C, IPFnet investigators (2013). Suspected acute exacerbation of idiopathic pulmonary fibrosis as an outcome measure in clinical trials. Respir Res.

[11] Ryerson CJ, Cottin V, Brown KK, Collard HR (2005). Acute exacerbation of idiopathic pulmonary fibrosis: shifting the paradigm. Eur Respir J.

[12] Papiris SA, Kagouridis K, Kolilekas L, Karakatsani A, Korbila I, Giouleka P, Papadaki G, Maniati M, Bouros D, Manali ED (2017). The new idiopathic pulmonary fibrosis acute exacerbations document: one step ahead but still suspended in the air. Am J Respir Crit Care Med.

[13] Schupp JC, Binder H, Jäger B, Cillis G, Zissel G, Müller-Quernheim J, Prasse A (2015). Macrophage activation in acute exacerbation of idiopathic pulmonary fibrosis. PLoS One.

[14] Belperio JA, Keane MP, Burdick MD, Londhe V, Xue YY, Li K, Phillips RJ, Strieter RM (2002). Critical role for CXCR2 and CXCR2 ligands during the pathogenesis of ventilator-induced lung injury. J Clin Invest.

[15] Donnelly SC, Strieter RM, Kunkel SL, Walz A, Robertson CR, Carter DC, Grant IS, Pollok AJ, Haslett C (1993). Interleukin-8 and development of adult respiratory distress syndrome in at-risk patient groups. Lancet.

[16] Hirani N, Antonicelli F, Strieter RM, Wiesener MS, Ratcliffe PJ, Haslett C, Donnelly SC (2001). The regulation of interleukin-8 by hypoxia in human macrophages—a potential role in the pathogenesis of the acute respiratory distress syndrome (ARDS). Mol Med.

[17] Mora AL, Torres-González E, Rojas M, Corredor C, Ritzenthaler J, Xu J, Roman J, Brigham K, Stecenko A (2006). Activation of alveolar macrophages via the alternative pathway in herpesvirus-induced lung fibrosis. Am J Respir Cell Mol Biol.

[18] Osterholzer JJ, Olszewski MA, Murdock BJ, Chen GH, Erb-Downward JR, Subbotina N, Browning K, Lin Y, Morey RE, Dayrit JK, Horowitz JC, Simon RH, Sisson TH (2013). Implicating exudate macrophages and Ly-6C(high) monocytes in CCR2-dependent lung fibrosis following gene-targeted alveolar injury. J Immunol.

[19] Prasse A, Pechkovsky DV, Toews GB, Jungraithmayr W, Kollert F, Goldmann T, Vollmer E, Müller-Quernheim J, Zissel G (2006). A vicious circle of alveolar macrophages and fibroblasts perpetuates pulmonary fibrosis via CCL18. Am J Respir Crit Care Med.

[20] Balestro E, Calabrese F, Turato G, Lunardi F, Bazzan E, Marulli G, Biondini D, Rossi E, Sanduzzi A, Rea F, Rigobello C, Gregori D, Baraldo S, Spagnolo P, Cosio MG, Saetta M (2006). Immune inflammation and disease progression in idiopathic pulmonary fibrosis. PLoS One.

[21] Papiris SA, Tomos IP, Karakatsani A, Spathis A, Korbila I, Analitis A, Kolilekas L, Kagouridis K, Loukides S, Karakitsos P, Manali ED. High levels of IL-6 and IL-8 characterize early-on Idiopathic pulmonary fibrosis acute exacerbations. Cytokine. 2017;102:168-72. 10.1016/j.cyto.2017.08.019 28847533

[22] Slutsky AS, Ranieri VM (2013). Ventilator-induced lung injury. N Engl J Med.

[23] Neto AS, Simonis FD, Barbas CS (2015). Lung-protective ventilation with low tidal volumes and the occurrence of pulmonary complications in patients without acute respiratory distress syndrome: a systematic review and individual patient data analysis. Crit Care Med.

[24] The Acute Respiratory Distress Syndrome Network (2000). Ventilation with lower tidal volumes as compared with traditional tidal volumes for acute lung injury and the acute respiratory distress syndrome. N Engl J Med.

[25] Petrucci N, De Feo C (2013). Lung protective ventilation strategy for the acute respiratory distress syndrome. Cochrane Database Syst Rev.

[26] Gattinoni L, Carlesso E, Caironi P (2002). Stress and strain within the lung. Curr Opin Crit Care.

[27] Protti A, Cressoni M, Santini A, Langer T, Mietto C, Febres D, Chierichetti M, Coppola S, Conte G, Gatti S, Leopardi O, Masson S, Lombardi L, Lazzerini M, Rampoldi E, Cadringher P, Gattinoni L (2011). Lung stress and strain during mechanical ventilation: any safe threshold?. Am J Respir Crit Care Med.

[28] Chiumello D, Carlesso E, Cadringher P, Caironi P, Valenza F, Polli F, Tallarini F, Cozzi P, Cressoni M, Colombo A, Marini JJ, Gattinoni L (2008). Lung stress and strain during mechanical ventilation for acute respiratory distress syndrome. Am J Respir Crit Care Med.

[29] Mauri T, Yoshida T, Bellani G, Goligher EC, Carteaux G, Rittayamai N, Mojoli F, Chiumello D, Piquilloud L, Grasso S, Jubran A, Laghi F, Magder S, Pesenti A, Loring S, Gattinoni L, Talmor D, Blanch L, Amato M, Chen L, Brochard L, Mancebo J, PLeUral pressure working Group (PLUG—Acute Respiratory Failure section of the European Society of Intensive Care Medicine) (2016). Esophageal and transpulmonary pressure in the clinical setting: meaning, usefulness and perspectives. Intensive Care Med.

[30] Pitsiou G, Trigonis I, Tsiata E, Kontou P, Manolakoglou N, Stanopoulos I, et al. Argyropoulou (Thessaloniki, Greece) outcome of patients with pulmonary fibrosis admitted to the ICU for acute respiratory failure. Eur Respir J. 2006;(28 Suppl).

[31] Pelosi P, Cereda M, Foti G, Giacomini M, Pesenti A (1995). Alterations of lung and chest wall mechanics in patients with acute lung injury: effects of positive end-expiratory pressure. Am J Respir Crit Care Med.

[32] Gattinoni L, Chiumello D, Carlesso E, Valenza F (2004). Bench-to-bedside review: chest wall elastance in acute lung injury/acute respiratory distress syndrome patients. Crit Care.

[33] Ranieri VM, Brienza N, Santostasi S, Puntillo F, Mascia L, Vitale N, Giuliani R, Memeo V, Bruno F, Fiore T, Brienza A, Slutsky AS (1997). Impairment of lung and chest wall mechanics in patients with acute respiratory distress syndrome: role of abdominal distension. Am J Respir Crit Care Med.

[34] Cressoni M, Cadringher P, Chiurazzi C, Amini M, Gallazzi E, Marino A, Brioni M, Carlesso E, Chiumello D, Quintel M, Bugedo G, Gattinoni L (2014). Lung inhomogeneity in patients with acute respiratory distress syndrome. Am J Respir Crit Care Med.

[35] Protti A, Andreis DT, Milesi M (2015). Lung anatomy, energy load, and ventilator-induced lung injury. Intensive Care Med Exp.

[36] Gattinoni L, Tonetti T, Cressoni M (2016). Ventilator-related causes of lung injury: the mechanical power. Intensive Care Med.

[37] Nava S, Rubini F (1999). Lung and chest wall mechanics in ventilated patients with end stage idiopathic pulmonary fibrosis. Thorax.

[38] Lutz D, Gazdhar A, Lopez-Rodriguez E, Ruppert C, Mahavadi P, Günther A, Klepetko W, Bates JH, Smith B, Geiser T, Ochs M, Knudsen L (2015). Alveolar derecruitment and collapse induration as crucial mechanisms in lung injury and fibrosis. Am J Respir Cell Mol Biol.

[39] Papiris SA, Manali ED, Kolilekas L (2010). Clinical review: Idiopathic pulmonary fibrosis acute exacerbations—unravelling Ariadne’s thread. Crit Care.

[40] Kondoh Y, Cottin V, Brown KK (2017). Recent lessons learned in the management of acute exacerbation of idiopathic pulmonary fibrosis. Eur Respir Rev.

[41] Aliberti S, Messinesi G, Gamberini S, Maggiolini S (2014). Non-invasive mechanical ventilation in patients with diffuse interstitial lung diseases. BMC Pulm Med.

[42] Papiris SA, Kagouridis K, Kolilekas L, Bouros D, Manali ED (2014). Idiopathic pulmonary fibrosis acute exacerbations: where are we now?. Expert Rev Respir Med.

[43] Bellani G, Laffey JG, Pham T, Fan E, Brochard L, Esteban A, Gattinoni L, van Haren F, Larsson A, McAuley DF, Ranieri M, Rubenfeld G, Thompson BT, Wrigge H, Slutsky AS, Pesenti A, LUNG SAFE Investigators; ESICM Trials Group (2016). Epidemiology, patterns of care, and mortality for patients with acute respiratory distress syndrome in intensive care units in 50 countries. JAMA.

[44] Papiris SA, Maniati M, Kagouridis K, Kolilekas L, Triantafillidou C, Papadaki G, et al. Idiopathic pulmonary fibrosis acute exacerbations: time to “turn the screw”. Cambridge: Treatment Strategies Cambridge Research Centre; 2014. pp. 47-9.

[45] Terragni PP, Rosboch G, Tealdi A (2007). Tidal hyperinflation during low tidal volume ventilation in acute respiratory distress syndrome. Am J Respir Crit Care Med.

[46] Papazian L, Forel JM, Gacouin A, Penot-Ragon C, Perrin G, Loundou A, Jaber S, Arnal JM, Perez D, Seghboyan JM, Constantin JM, Courant P, Lefrant JY, Guérin C, Prat G, Morange S, Roch A (2010). ACURASYS Study Investigators. Neuromuscular blockers in early acute respiratory distress syndrome. N Engl J Med.

[47] Fernández-Pérez ER, Yilmaz M, Jenad H, Daniels CE, Ryu JH, Hubmayr RD, Gajic O (2008). Ventilator settings and outcome of respiratory failure in chronic interstitial lung disease. Chest.

[48] Kallet RH (2015). A comprehensive review of prone position in ARDS. Respir Care.

[49] Blanch L, Mancebo J, Perez M, Martinez M, Mas A, Betbese AJ, Joseph D, Ballús J, Lucangelo U, Bak E (1997). Short-term effects of prone position in critically ill patients with acute respiratory distress syndrome. Intensive Care Med.

[50] Frat JP (2015). High-flow oxygen through nasal cannula in acute hypoxemic respiratory failure. N Engl J Med.

[51] Guérin C, Reignier J, Richard JC, Beuret P, Gacouin A, Boulain T, Mercier E, Badet M, Mercat A, Baudin O, Clavel M, Chatellier D, Jaber S, Rosselli S, Mancebo J, Sirodot M, Hilbert G, Bengler C, Richecoeur J, Gainnier M, Bayle F, Bourdin G, Leray V, Girard R, Baboi L, Ayzac L, PROSEVA Study Group (2013). Prone positioning in severe acute respiratory distress syndrome. N Engl J Med.

[52] Nakos G, Tsangaris I, Kostanti E, Nathanail C, Lachana A, Koulouras V, Kastani D (2000). Effect of the prone position on patients with hydrostatic pulmonary edema compared with patients with acute respiratory distress syndrome and pulmonary fibrosis. Am J Respir Crit Care Med.

[53] Putensen C, Muders T, Varelmann D, Wrigge H (2006). The impact of spontaneous breathing during mechanical ventilation. Curr Opin Crit Care.

[54] Yoshida T, Uchiyama A, Matsuura N, Mashimo T, Fujino Y (2013). The comparison of spontaneous breathing and muscle paralysis in two different severities of experimental lung injury. Crit Care Med.

[55] Carvalho NC, Güldner A, Beda A (2014). Higher levels of spontaneous breathing reduce lung injury in experimental moderate acute respiratory distress syndrome. Crit Care Med.

[56] Brochard L, Slutsky A, Pesenti A (2016). Mechanical ventilation to minimize progression of lung injury in acute respiratory failure. Am J Respir Crit Care Med.

[57] Bellani G, Grasselli G, Teggia-Droghi M, Mauri T, Coppadoro A, Brochard L, Pesenti A (2016). Do spontaneous and mechanical breathing have similar effects on average transpulmonary and alveolar pressure? A clinical crossover study. Crit Care.

[58] Yoshida T, Fujino Y, Amato MB, Kavanagh BP (2016). Fifty years of research in ARDS. Spontaneous breathing during mechanical ventilation—risks, mechanisms & management. Am J Respir Crit Care Med.

[59] Yoshida T, Torsani V, Gomes S, De Santis RR, Beraldo MA, Costa EL, Tucci MR, Zin WA, Kavanagh BP, Amato MB (2013). Spontaneous effort causes occult pendelluft during mechanical ventilation. Am J Respir Crit Care Med.

[60] Horio Y (2016). High-flow nasal cannula oxygen therapy for acute exacerbation of interstitial pneumonia: a case series. Respir Investig.

[61] Brodie D, Bacchetta M (2011). Extracorporeal membrane oxygenation for ARDS in adults. N Engl J Med.

[62] Neto SA, for the ReVA Research Network and the PROVE Network Investigators (2016). Associations between ventilator settings during extracorporeal membrane oxygenation for refractory hypoxemia and outcome in patients with acute respiratory distress syndrome: a pooled individual patient data analysis: mechanical ventilation during ECMO. Intensive Care Med.

[63] Singer JP, Blanc PD, Hoopes C, Golden JA, Koff JL, Leard LE, Cheng S, Chen H (2011). The impact of pretransplant mechanical ventilation on short- and long-term survival after lung transplantation. Am J Transplant.

[64] Fuehner T, Kuehn C, Hadem J, Wiesner O, Gottlieb J, Tudorache I, Olsson KM, Greer M, Sommer W, Welte T, Haverich A, Hoeper MM, Warnecke G (2012). Extracorporeal membrane oxygenation in awake patients as bridge to lung transplantation. Am J Respir Crit Care Med.

[65] Chiumello D, Coppola S, Froio S, Colombo A, Del Sorbo L (2015). Extracorporeal life support as bridge to lung transplantation: a systematic review. Crit Care.

[66] Meduri GU, Headley S, Kohler G, Stentz F, Tolley E, Umberger R, Leeper K (1995). Persistent elevation of inflammatory cytokines predicts a poor outcome in ARDS. Plasma IL-1 beta and IL-6 levels are consistent and efficient predictors of outcome over time. Chest.

[67] Steinberg KP, Hudson LD, Goodman RB, Hough CL, Lanken PN, Hyzy R, Thompson BT, Ancukiewicz M (2006). National Heart, Lung, and Blood Institute Acute Respiratory Distress Syndrome (ARDS) Clinical Trials Network. Efficacy and safety of corticosteroids for persistent acute respiratory distress syndrome. N Engl J Med.

[68] Tajima S, Oshikawa K, Tominaga S, Sugiyama Y (2003). The increase in serum soluble ST2 protein upon acute exacerbation of idiopathic pulmonary fibrosis. Chest.

[69] Juarez MM, Chan AL, Norris AG, Morrissey BM, Albertson TE (2015). Acute exacerbation of idiopathic pulmonary fibrosis—a review of current and novel pharmacotherapies. J Thorac Dis.

[70] Papiris SA, Manali ED, Kolilekas L, Triantafillidou C, Tsangaris I, Kagouridis K (2012). Steroids in idiopathic pulmonary fibrosis acute exacerbation: defenders or killers?. Am J Respir Crit Care Med.

[71] Cottin V, Crestani B, Valeyre D, Wallaert B, Cadranel J, Dalphin JC, Delaval P, Israel-Biet D, Kessler R, Reynaud-Gaubert M, Aguilaniu B, Bouquillon B, Carré P, Danel C, Faivre JB, Ferretti G, Just N, Kouzan S, Lebargy F, Marchand-Adam S, Philippe B, Prévot G, Stach B, Thivolet-Béjui F, Cordier JF, French National Reference Centre; Network of Competence Centres for Rare Lung Diseases (2014). Diagnosis and management of idiopathic pulmonary fibrosis: French practical guidelines. Eur Respir Rev.

[72] Morawiec E, Tillie-Leblond I, Pansini V, Salleron J, Remy-Jardin M, Wallaert B (2011). Exacerbations of idiopathic pulmonary fibrosis treated with corticosteroids and cyclophosphamide pulses. Eur Respir J.

[73] Papiris SA, Kagouridis K, Kolilekas L, Papaioannou AI, Roussou A, Triantafillidou C, Baou K, Malagari K, Argentos S, Kotanidou A, Karakatsani A, Manali ED (2015). Survival in idiopathic pulmonary fibrosis acute exacerbations: the non-steroid approach. BMC Pulm Med.

[74] Richeldi L, Costabel U, Selman M, Kim DS, Hansell DM, Nicholson AG, Brown KK, Flaherty KR, Noble PW, Raghu G (2011). Efficacy of a tyrosine kinase inhibitor in idiopathic pulmonary fibrosis. N Engl J Med.

[75] Richeldi L, du Bois RM, Raghu G, Azuma A, Brown KK, Costabel U, Cottin V, Flaherty KR, Hansell DM, Inoue Y, Kim DS, Kolb M, Nicholson AG, Noble PW, Selman M, Taniguchi H, Brun M, Le Maulf F, Girard M, Stowasser S, Schlenker-Herceg R, Disse B, Collard HR, INPULSIS Trial Investigators (2014). Efficacy and safety of nintedanib in idiopathic pulmonary fibrosis. N Engl J Med.

[76] Azuma A, Nukiwa T, Tsuboi E, Suga M, Abe S, Nakata K, Taguchi Y, Nagai S, Itoh H, Ohi M (2005). Double-blind, placebo-controlled trial of pirfenidone in patients with idiopathic pulmonary fibrosis. Am J Respir Crit Care Med.

[77] Taniguchi H, Ebina M, Kondoh Y, Ogura T, Azuma A, Suga M, Taguchi Y, Takahashi H, Nakata K, Sato A, Takeuchi M, Raghu G, Kudoh S, Nukiwa T (2010). Pirfenidone Clinical Study Group in Japan. Pirfenidone in idiopathic pulmonary fibrosis. Eur Respir J.

[78] Lee JS, Collard HR, Anstrom KJ, Martinez FJ, Noth I, Roberts RS, Yow E, Raghu G, IPFnet Investigators (2013). Anti-acid treatment and disease progression in idiopathic pulmonary fibrosis: an analysis of data from three randomised controlled trials. Lancet Respir Med.

[79] Akira M, Hamada H, Sakatani M, Kobayashi C, Nishioka M, Yamamoto S (1997). CT findings during phase of accelerated deterioration in patients with idiopathic pulmonary fibrosis. AJR Am J Roentgenol.

[80] Al-Hameed FM, Sharma S (2004). Outcome of patients admitted to the intensive care unit for acute exacerbation of idiopathic pulmonary fibrosis. Can Respir J.

[81] Suzuki H, Sekine Y, Yoshida S, Suzuki M, Shibuya K, Yonemori Y, Hiroshima K, Nakatani Y, Mizuno S, Takiguchi Y (2011). Risk of acute exacerbation of interstitial pneumonia after pulmonary resection for lung cancer in patients with idiopathic pulmonary fibrosis based on preoperative high-resolution computed tomography. Surg Today.

[82] Tachikawa R, Tomii K, Ueda H, Nagata K, Nanjo S, Sakurai A, Otsuka K, Kaji R, Hayashi M, Katakami N (2012). Clinical features and outcome of acute exacerbation of interstitial pneumonia: collagen vascular diseases-related versus idiopathic. Respiration.

[83] Akira M, Kozuka T, Yamamoto S, Sakatani M (2008). Computed tomography findings in acute exacerbation of idiopathic pulmonary fibrosis. Am J Respir Crit Care Med.

[84] Fujimoto K, Taniguchi H, Johkoh T, Kondoh Y, Ichikado K, Sumikawa H, Ogura T, Kataoka K, Endo T, Kawaguchi A (2012). Acute exacerbation of idiopathic pulmonary fibrosis: high-resolution CT scores predict mortality. Eur Radiol.

[85] Yokoyama T, Kondoh Y, Taniguchi H, Kataoka K, Kato K, Nishiyama O, Kimura T, Hasegawa R, Kubo K (2010). Noninvasive ventilation in acute exacerbation of idiopathic pulmonary fibrosis. Intern Med.

[86] Homma S, Sakamoto S, Kawabata M, Kishi K, Tsuboi E, Motoi N, Yoshimura K (2005). Cyclosporin treatment in steroid-resistant and acutely exacerbated interstitial pneumonia. Intern Med.

[87] Inase N, Sawada M, Ohtani Y, Miyake S, Isogai S, Sakashita H, Miyazaki Y, Yoshizawa Y (2003). Cyclosporin A followed by the treatment of acute exacerbation of idiopathic pulmonary fibrosis with corticosteroid. Intern Med.

[88] Sakamoto S, Homma S, Miyamoto A, Kurosaki A, Fujii T, Yoshimura K (2010). Cyclosporin A in the treatment of acute exacerbation of idiopathic pulmonary fibrosis. Intern Med.

[89] Abe S, Hayashi H, Seo Y, Matsuda K, Kamio K, Saito Y, Usuki J, Azuma A, Kudo S, Gemma A (2011). Reduction in serum high mobility group box-1 level by polymyxin B-immobilized fiber column in patients with idiopathic pulmonary fibrosis with acute exacerbation. Blood Purif.

[90] Abe S, Azuma A, Mukae H, Ogura T, Taniguchi H, Bando M, Sugiyama Y (2012). Polymyxin B-immobilized fiber column (PMX) treatment for idiopathic pulmonary fibrosis with acute exacerbation: a multicenter retrospective analysis. Intern Med.

[91] Oishi K, Mimura-Kimura Y, Miyasho T, Aoe K, Ogata Y, Katayama H, Murata Y, Ueoka H, Matsumoto T, Mimura Y (2013). Association between cytokine removal by polymyxin B hemoperfusion and improved pulmonary oxygenation in patients with acute exacerbation of idiopathic pulmonary fibrosis. Cytokine.

[92] Seo Y, Abe S, Kurahara M, Okada D, Saito Y, Usuki J, Azuma A, Koizumi K, Kudoh S (2006). Beneficial effect of polymyxin B-immobilized fiber column (PMX) hemoperfusion treatment on acute exacerbation of idiopathic pulmonary fibrosis. Intern Med.

[93] Tachibana K, Inoue Y, Nishiyama A, Sugimoto C, Matsumuro A, Hirose M, Kitaichi M, Akira M, Arai T, Hayashi S (2011). Polymyxin-B hemoperfusion for acute exacerbation of idiopathic pulmonary fibrosis: serum IL-7 as a prognostic marker. Sarcoidosis Vasc Diffuse Lung Dis.

[94] Donahoe M, Valentine VG, Chien N, Gibson KF, Raval JS, Saul M, Xue J, Zhang Y, Duncan SR (2015). Autoantibody-targeted treatments for acute exacerbations of idiopathic pulmonary fibrosis. PLoS One..

[95] Horita N, Akahane M, Okada Y, Kobayashi Y, Arai T, Amano I, Takezawa T, To M, To Y (2011). Tacrolimus and steroid treatment for acute exacerbation of idiopathic pulmonary fibrosis. Intern Med.

[96] Kataoka K, Taniguchi H, Kondoh Y, Nishiyama O, Kimura T, Matsuda T, Yokoyama T, Sakamoto K, Ando M (2015). Recombinant human thrombomodulin in acute exacerbation of idiopathic pulmonary fibrosis. Chest.

[97] Tsushima K, Yamaguchi K, Kono Y, Yokoyama T, Kubo K, Matsumura T, Ichimura Y, Abe M, Terada J, Tatsumi K (2014). Thrombomodulin for acute exacerbations of idiopathic pulmonary fibrosis: a proof of concept study. Pulm Pharmacol Ther.

[98] Isshiki T, Sakamoto S, Kinoshita A, Sugino K, Kurosaki A, Homma S (2015). Recombinant human soluble thrombomodulin treatment for acute exacerbation of idiopathic pulmonary fibrosis: a retrospective study. Respiration.

[99] Kase Y, Obata T, Okamoto Y (2008). Removal of 2-arachidonylglycerol by direct hemoperfusion therapy with polymyxin B immobilized fibers benefits patients with septic shock. Ther Apher Dial.

[100] Cruz DN, Antonelli M, Fumagalli R (2009). Early use of polymyxin B hemoperfusion in abdominal septic shock: the EUPHAS randomized controlled trial. JAMA.

[101] Nakamura T, Kawagoe Y, Matsuda T (2004). Effect of polymyxin B-immobilized fiber on blood metalloproteinase-9 and tissue inhibitor of metalloproteinase-1 levels in acute respiratory distress syndrome patients. Blood Purif.

[102] Enomoto N (2015). Treatment of acute exacerbation of idiopathic pulmonary fibrosis with direct hemoperfusion using a polymyxin B-immobilized fiber column improves survival. BMC Pulm Med.

[103] Keating D, Levvey B, Kotsimbos T (2009). Lung transplantation in pulmonary fibrosis: challenging early outcomes counterbalanced by surprisingly good outcomes beyond 15 years. Transplant Proc.

[104] Orsini B, Sage E, Olland A (2014). High-emergency waiting list for lung transplantation: early results of a nation-based study. Eur J Cardiothorac Surg.

[105] Gottlieb J, Warnecke G, Hadem J, Dierich M (2012). Outcome of critically ill lung transplant candidates on invasive respiratory support. Intensive Care Med.

[106] Molina-Molina M, Badia JR, Marín-Arguedas A, Xaubet A (2003). Outcomes and clinical characteristics of patients with pulmonary fibrosis and respiratory failure admitted to an intensive care unit. A study of 20 cases. Med Clin.

[107] Stern JB, Mal H, Groussard O, Brugière O, Marceau A, Jebrak G, Fournier M (2001). Prognosis of patients with advanced idiopathic pulmonary fibrosis requiring mechanical ventilation for acute respiratory failure. Chest.

[108] Blivet S, Philit F, Sab JM, Langevin B, Paret M, Guérin C, Robert D (2001). Outcome of patients with idiopathic pulmonary fibrosis admitted to the ICU for respiratory failure. Chest.

[109] Saydain G, Islam A, Afessa B, Ryu JH, Scott JP, Peters SG (2002). Outcome of patients with idiopathic pulmonary fibrosis admitted to the intensive care unit. Am J Respir Crit Care Med.

[110] Fumeaux T, Rothmeier C, Jolliet P (2001). Outcome of mechanical ventilation for acute respiratory failure in patients with pulmonary fibrosis. Intensive Care Med.

[111] Kim DS, Park JH, Park BK, Lee JS, Nicholson AG, Colby T (2006). Acute exacerbation of idiopathic pulmonary fibrosis: frequency and clinical features. Eur Respir J.

[112] Rangappa P, Moran JL (2009). Outcomes of patients admitted to the intensive care unit with idiopathic pulmonary fibrosis. Crit Care Resusc.

[113] Fernández-Pérez ER, Yilmaz M, Jenad H, Daniels CE (2008). Ventilator Settings and Outcome of Respiratory Failure in Chronic Interstitial Lung Disease. Chest.

[114] Mollica C, Paone G, Conti V, Ceccarelli V (2010). Mechanical Ventilation in Patients with End-Stage Idiopathic Pulmonary Fibrosis. Respir.

[115] Gungor G, Tatar D, Salturk C, Cimen P (2013). Why Do Patients With Interstitial Lung Diseases Fail in the ICU? A 2-Center Cohort Study. Respir Care.

[116] Vianello A, Arcaro G, Battistella L, Pipitone E (2014). Noninvasive ventilation in the event of acute respiratory failure in patients with idiopathic pulmonary fibrosis. J Crit Care.

[117] Gaudry S, Vincent S, Rabbat A, Nunes H (2014). Invasive mechanical ventilation in patients with fibrosing interstitial pneumonia. J Thorac Cardiovasc Surg.

[118] Aliberti S, Messinesi G, Gamberini S, Maggiolini S, et al. Non-invasive mechanical ventilation in patients with diffuse interstitial lung diseases. BMC Pulm Med. 2014;14(1). 10.1186/1471-2466-14-194 PMC4269964 25476922

